# Structural Analysis of microRNA-Target Interaction by Sequential Seed Mutagenesis and Stem-Loop 3' RACE

**DOI:** 10.1371/journal.pone.0081427

**Published:** 2013-11-25

**Authors:** Marc Bohmer, Jutta Sharbati, Jennifer zur Bruegge, Ralf Einspanier, Soroush Sharbati

**Affiliations:** Institute of Veterinary-Biochemistry, Freie Universität Berlin, Berlin, Germany; The University of Tokyo, Japan

## Abstract

**Background:**

As a consequence of recent RNAseq efforts, miRNAomes of diverse tissues and species are available. However, most interactions between microRNAs and regulated mRNAs are still to be deciphered. While in silico analysis of microRNAs results in prediction of hundreds of potential targets, bona-fide interactions have to be verified e.g. by luciferase reporter assays using fused target sites as well as controls incorporating mutated seed sequences. The aim of this study was the development of a straightforward approach for sequential mutation of multiple target sites within a given 3’ UTR.

**Methodology/Principal Findings:**

The established protocol is based on Seed Mutagenesis Assembly PCR (SMAP) allowing for rapid identification of microRNA target sites. Based on the presented approach, we were able to determine the transcription factor NKX3.1 as a genuine target of miR-155. The sequential mutagenesis of multiple microRNA target sites was examined by miR-29a mediated CASP7 regulation, which revealed one of two predicted target sites as the predominant site of interaction. Since 3’ UTR sequences of non-model organisms are either lacking in databases or computationally predicted, we developed a Stem-Loop 3’ UTR RACE PCR (SLURP) for efficient generation of required 3’ UTR sequence data. The stem-loop primer allows for first strand cDNA synthesis by nested PCR amplification of the 3’ UTR. Besides other applications, the SLURP method was used to gain data on porcine CASP7 3’UTR evaluating evolutionary conservation of the studied interaction.

**Conclusions/Significance:**

Sequential seed mutation of microRNA targets based on the SMAP approach allows for rapid structural analysis of several target sites within a given 3’ UTR. The combination of both methods (SMAP and SLURP) enables targeted analysis of microRNA binding sites in hitherto unknown mRNA 3’ UTRs within a few days.

## Introduction

Over the course of the last two decades the importance of microRNAs (miRNAs) in regulating crucial biological processes both in the animal and plant kingdom is recognised. In particular, the invention and application of next generation sequencing have led to the discovery of hundreds of miRNAs in various animals including humans and mice [1-3]. MiRNAs, which have been identified in numerous taxa, not only regulate animal ontogeny, but their aberrant expression leads to severe diseases such as cancer or immune disorders. The next step to unravelling their role is to elicit how novel and known molecules function in different cellular contexts. In general, miRNAs regulate gene expression by affecting protein synthesis either via translational repression or degradation of mRNA after deadenylation [4]. Animal miRNAs are expressed as single transcripts or as clusters in a polycistronic way. After successive processing by the nucleases Drosha and Dicer, the active RNA induced silencing complex (RISC) is formed containing the mature miRNA, which with few exceptions exhibits imperfect complementarity to the target site in the mRNA. A general rule for miRNA binding and activity is the formation of a perfect Watson-Crick hybrid between the miRNA 5’ nucleotides 2-8 (referred to as the “seed” of miRNA) and the target site of the mRNA generally located in the mRNA 3’ UTR. Furthermore, advanced miRNA activity is observed for molecules possessing an adenosine across position 1 and adenosine or uridine across position 9 [4]. Another rule for canonical miRNA binding is that bulges or mismatches are needed in the central region of miRNAs followed by target complementarity at the 3’ end [5]. However, several studies have suggested that non-canonical seed binding also leads to miRNA mediated silencing [6,7].

Apart from the understanding of miRNA biogenesis and its regulation, identification of miRNA targets is key to unravelling mechanisms of miRNA function. However, based on both their small size and the incomplete miRNA-mRNA interaction, target prediction and analysis are very demanding and involved. As reviewed by Alexiou and colleagues [8], the development of numerous target prediction algorithms e.g. Target Scan [9], DIANA-microT [10] or RNAhybrid [11] has helped to rapidly identify putative miRNA targets. For example, Target Scan prediction is based on the fact that many miRNAs are conserved among phylogenetically related animals and it seems highly probable that conserved and aligned seeds in several species point to a biologically functional miRNA-mRNA interaction. However, a typical search often results in the prediction of hundreds of targets. Subsequent RNAhybrid analysis, an algorithm which finds the energetically most favourable hybridisation sites of a miRNA in the mRNA 3’ UTR, is a useful tool for narrowing down the number of potential targets. On the other hand, while target site prediction for common model organisms such as humans or mice are in the majority of cases easy to perform, a lack of 3’ UTR sequence data of non-model organisms often hampers in silico prediction. Therefore and prior to prediction of potential target sites, 3’ RACE (Rapid Amplification of cDNA Ends) experiments have to be performed to determine sequences and follow-up experiments are required to verify the predicted targets and the biological functionality of the miRNA. Orom and Lund [12] have reviewed that the limitation of target prediction using e.g. mentioned programs relies on few established principles of miRNA biology not allowing to consider novel aspects of miRNA target recognition. They conclude that many predicted targets do not show regulation in validation experiments. Experimental target identification can be performed using indicative or straight approaches. Indicative experiments represent mainly high-throughput methods such as transcriptome analysis after miRNA overexpression using either synthetic miRNAs or inhibition by means of complementary oligonucleotides. These approaches allow drawing conclusion from the altered expression of affected genes and pathways and are mainly limited to mRNAs that are degraded by their targeting miRNAs. Immunoprecipitation of AGO proteins followed by microarray analysis or sequencing is a more straightforward approach to identify miRNA targets, however, involves experimental challenges and difficulties. Since the final outcome of miRNA action regardless of causing translational inhibition or mRNA degradation is the regulation of cellular protein concentration, proteome analysis seems to be another suitable direct approach giving comprehensive insight into miRNA target identification [12]. 

Nevertheless, by either applying an indicative or straight approach, generated results are mostly validated by means of luciferase reporter gene assays where mutated seeds serve as controls. Here we present a convenient strategy to identify miRNA targets based on a modular approach to sequentially mutate multiple seeds within a given 3’ UTR. Furthermore, we present a new Stem-Loop 3' UTR RACE-PCR for rapid identification of unknown 3’ UTR sequences in non-model animals. Taken together, presented methods provide a straightforward strategy for functional and structural analysis of mRNA-miRNA interactions as examined for several candidates. The developed approach for miRNA target site mutagenesis allows for structural analysis of multiple miRNA target sites of a given mRNA. This innovation is of great interest, since effective regulation of a particular mRNA requires multiple target sites of the same or different microRNAs [5]. The integrative strength of presented technologies relies on combination of 3’ UTR sequence identification and functional target analysis in hitherto unknown data, facilitating discovery of regulative pathways also in non-model organisms.

## Materials and Methods

### Cell lines, isolation of total RNA and reverse transcription

The human cervix carcinoma cell line HeLa (ATCC No. CCL-2) and human monocytic cell line U937 (DSMZ No. ACC 5) were maintained and passaged twice weekly in RPMI 1640 (Biochrom AG) supplemented with 10% fetal bovine serum superior (Biochrom AG) and 10 µg/ml Gentamicin (Biochrom AG). Cultivation of cells was performed in 75 cm^2^ flasks (Greiner Bio-One GmbH) at 37 °C and 5% CO_2_.

Total RNA was isolated using the mirVana miRNA Isolation Kit (Life Technologies), according to the manufacturer’s protocol. The RNA quality and quantity of all samples were assessed as described previously [3]. Reverse transcription of total RNA was performed as described earlier [13]. Briefly, 1 µg RNA was treated with 1 U RNase free DNase (Thermo) in 10 µl total volume according to manufacturer’s protocol. Subsequently, the treated RNA was reverse transcribed in 20 µl total volume using 0.2 µg random hexamers, 200 µM of each dNTP and 200 U RevertAid™ Reverse Transcriptase (Thermo) following the manufacturer’s protocol. Reverse Transcriptase negative samples served as control for absence of contaminating DNA.

### Statistical Analysis

All experiments were performed considering at least three biological replicates each measured in triplicates. Unpaired t tests were performed and two-tailed P values were calculated using GraphPad Prism version 6.00 for Windows, GraphPad Software, La Jolla California USA, www.graphpad.com. Asterisks in figures summarise P values (*: P < 0.05; **: P < 0.01; ***: P < 0.001; ****: P < 0.0001).

### Nucleofection and luciferase reporter gene assays

For RNA as well as protein isolation after RNAi, cell lines were transfected employing the Nucleofector Technology (Lonza AG) together with 1 x 10^6^ cells and 100 pmol of miRNA mimics, inhibitors or controls according to the manufacturer’s instructions. Following interfering molecules were used in this study: Pre-miR™ miR-155 and 29a Precursors, miRVana miR-155 inhibitor (Life Technologies), NKX3.1 ON-TARGETplus SMARTpool – Human and non targeting siRNA control (Thermo). Samples for RNA and protein analysis were taken at 24 and 48 hours after nucleofection, respectively.

For combined detection of *Gaussia* as well as *Cypridina* luciferase activity, nucleofection was performed as described earlier [14] using 5 x 10^5^ – 1 x 10^6^ HeLa using 0.9 - 1.8 µg reporter plasmid (pTKGluc derivatives, NEB GmbH), 100 - 200 ng normalisation plasmid (pTKCluc, NEB GmbH) and 100 pmol synthetic miRNA according to the manufacturer’s instructions. Luciferase activity was determined using respective Biolux Assay Kits (NEB GmbH) and white 96 well microplates (Greiner Bio-One GmbH) together with the automated luminometer FLUOstar OPTIMA (BMG Labtech). 

### mRNA degradation assay

Cells were transfected using nonsense synthetic miRNAs, mimics, inhibitors or siRNAs, respectively. At 24 h as well as 48 h post transfection cells were washed once with ice-cold PBS and RNA was isolated and reverse transcribed. Target gene specific RT-qPCR assays were performed as described earlier [14] using the oligonucleotides given in table 1. mRNA levels were calculated relative to the nonsense transfected controls. 

**Table 1:** Oligonucleotides used in this work.

Name	Sequence (5' - 3')
SMAP Primers	
NotIhSHIP1-3UTRfw	atgcggccgccccagctcgctcttggtact
XbaIhSHIP1-3UTRrev	ggcgtctagagaggccagatctctccactg
hSHIP1 SDM f rev	gcactgggcttcgattagtcttcacccctccg
hSHIP SDM r fw	cggaggggtgaagactaatcgaagcccagtgc
NotIhCASP7-3UTR fw	tagcggccgcgctgagaagcaatgggtcac
XhoIhCASP7-3UTR rev	ggctcgagaggaattaagcaaccacatttt
hCASP7 SDM2 f rev	tcaagaattcaaaagaggtgtttggtgtgaaaacaaagtgcca
hCASP7 SDM2 r fw	tggcactttgttttcacaccaaacacctcttttgaattcttga
hCASP7 SDM1 f rev	ttcagagaacaaaagaggcgtgtggaaagtttcttccctg
hCASP7 SDM1 r fw	cagggaagaaactttccacacgcctcttttgttctctgaa
NotIhNKX3-3UTR fw	tagcggccgcctgctaggggctgttgcatt
XbaIhNKX3-3UTR rev	gccgtctagacgctgtgttcttcctctgtg
hNKX3 SDM f rev	ggatttgggatagctctgattgtatttttaaagg
hNKX3 SDM r fw	cctttaaaaatacaatcagagctatcccaaatcc
SLURP Primers	
SLURP rt	gtcctccctcccttacggttgctcatcggagaaagggagggaggacgatgttttttttttttttttttttvn
fw i	acggttgctcatcggaga
fw ii	aagggagggaggacgatg
sscDHH rev i	atgcggttctggagagtcac
sscDHH rev ii	tcggttcctctatcgtttgg
sscSELPLG rev i	gccaccaccaatggagtc
sscSELPLG rev ii	gaaggcagagcctcagaagg
sscTGFBR3rev i	gttcgcagcgtttgtgat
sscTGFBR3rev ii	ggggccttgtggtacatct
sscCASP7rev i	tgtattgcaggcagtgggta
sscCASP7fw ii	tgccccttaggtgttgtagg
mRNA degradation assay	
hsaNKX3-1 fw	agaaaggcacttggggtctt
hsaNKX3-1 rev	tccgtgagcttgaggttctt
hsaGAPDH fw	ccatcttccaggagcgagat
hsaGAPDH rev	ctaagcagttggtggtgcag

### Protein isolation and Western Blot

Protein was isolated using RIPA Buffer (Cell Signaling Technology) and according to the manufacturer’s protocol. Western Blots were performed applying 15 µg of protein extracts as described earlier [13] using the primary mouse anti-NKX3.1 (No. ab55781, purchased from Abcam) at 1:500 dilution and the secondary ECL Anti-mouse IgG antibody (No. NA9310V, purchased from GE Healthcare) at 1:5000 dilution. GAPDH was detected as a reference protein using the primary mouse anti-GAPDH (1D4) (No. NB300-221, purchased from Novus Biologicals) at 1:20.000 dilution and the same secondary antibody mentioned above at 1:40.000 dilution.

### Oligonucleotides for PCR

All PCR oligonucleotides used in this work were synthesised by Metabion AG and Sigma Aldrich (table 1). 

### Seed Mutagenesis Assembly PCR (SMAP)

**Figure 1:**
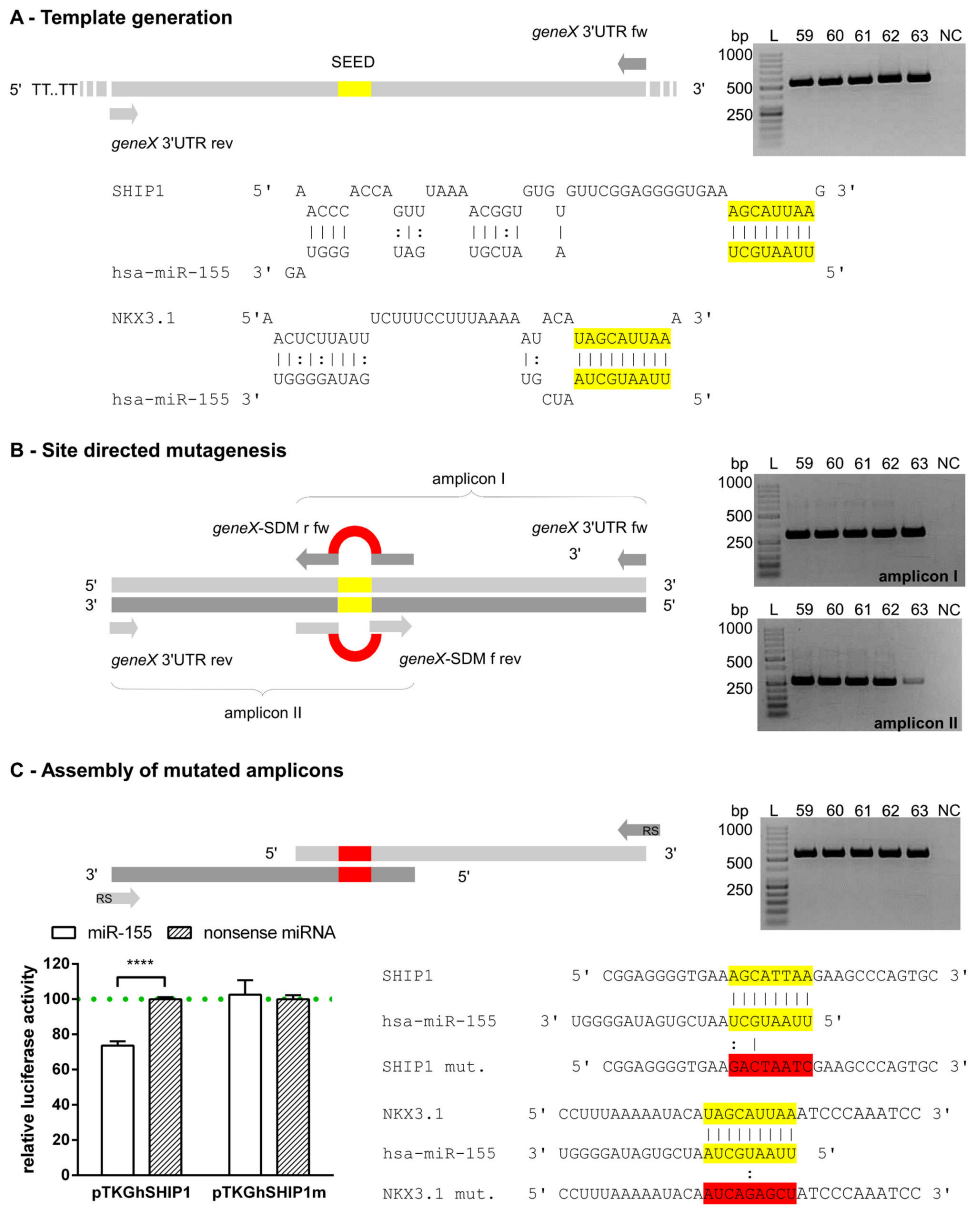
Concept of Seed Mutagenesis Assembly PCR (SMAP). Figure shows seed mutagenesis of miR-155 targets SHIP1 and NKX3.1. Gel images show amplicons obtained after each step applying a gradient PCR, while NC represents the no template control at the lowest annealing temperature. Section A: 3’ UTR amplicons were generated harbouring the predicted target site. Section B: Oligonucleotides were used for seed mutagenesis and generation of overlapping termini that possessed approximately 12 nucleotides at the 3’ as well as 5’ ends flanking the mutated site. Section C: Assembly PCR of amplicons with mutated termini provided 3’ UTRs including the mutated seed. The amplicon was fused to a luciferase gene for reporter gene assays. Relative luciferase activity (Luc _Gaussia_ : Luc _Cypridina_) was determined using a miR-155 mimic compared with a nonsense miRNA control together with the mutated seed (pTKGhSHIP1m) as well as wild type control (pTKGhSHIP1) . The columns show means of normalised luciferase activity of three biological replicates each measured in triplicates while error bars show the standard deviation. Asterisks indicate statistical significance between samples (****: P < 0.001, unpaired t test).

Templates for mutagenesis of target sites of the gene of interest were generated using 2 µl of reverse transcribed cDNA with 10 pmol of each primer (Figure 1; '*geneX* 3'UTR fw and rev'), 1 U Immolase DNA Polymerase (Bioline GmbH), 3 mM MgCl2, 0.2 mM dNTPs and 10x buffer in 25 µl total volume. The reaction was started at 95 °C for 8 min, followed by 40 cycles with 45 s at 95 °C, 15 s using a temperature gradient (59, 60, 61, 62 and 63 °C) and 30 s at 72 °C followed by a terminal step at 72 °C for 5 min. 

Site directed mutagenesis of unique or multiple miRNA target sites was achieved by applying primers with mutated seed sequences in PCR reactions providing two (considering a unique target site) or five (considering two target sites) partly overlapping amplicons with mutated seeds. Amplicon assembly resulted in recovery of full length 3’ UTR including the mutated site. Site directed mutagenesis of each of the flanks was performed using 0.05-0.1 pmol of gel purified template with 10 pmol of each primer (Figure 1; '*geneX* 3'UTR fw' and '*geneX* SDM f rev' for amplicon I or '*geneX* 3'UTR rev' and '*geneX* SDM r fw' for amplicon II), 1 U Immolase DNA Polymerase, 3 mM MgCl2, 0.2 mM dNTPs and 10x buffer in 25 µl total volume. After the initial step at 95 °C for 8 min, the first 10 cycles were run for 20 s at 95 °C, 10 s using a temperature gradient (54, 55, 56, 57 and 58 °C) and +dT 0.5 °C per cycle followed by 15 s at 72 °C. The next 30 cycles were conducted according following scheme: 20 s at 95 °C 10 s using a temperature gradient (59, 60, 61, 62 and 63 °C) and 15 s at 72 °C followed by a terminal step at 72 °C for 2 min.

Amplicon assembly of unique as well as multiple mutated sites was performed in a reaction consisting of two successive rounds. First, equimolar amounts of gel purified amplicons with overlapping termini were employed for template generation. Assembly PCR was performed using 0.5 pmol of each amplicon with 1 U Immolase DNA Polymerase, 3 mM MgCl2, 0.2 mM dNTPs and 10x buffer in 25 µl total volume. The assembly was started at 95 °C for 8 min, followed by 10 cycles with 30 s at 95 °C, 15 s using a temperature gradient (59, 60, 61, 62 and 63 °C) and 30 s at 72 °C. The reaction was cooled down to 4 °C. The amplification of the assembled 3’ UTR harbouring the mutated target site was achieved by adding 10 pmol of each terminal primer providing the restriction sites (RS) for cloning (table 1; RS-geneX 3UTRfw, RS geneX-3UTRrev) with 1 U Immolase DNA Polymerase, 3 mM MgCl2, 0.2 mM dNTPs and 10x buffer and bringing the total reaction volume to 50 µl. The second round was started at 95 °C for 8 min, followed by 25 cycles with 30 s at 95 °C, 15 s at 63 °C and 30 s at 72 °C followed by a terminal step at 72 °C for 2 min. 

The mutated 3’ UTR representing the experimental sample and wild type 3’ UTR serving as a control were digested with respective enzymes and cloned in pTKGluc (NEB GmbH) as described earlier [14], resulting in generation of following plasmids: pTKGhSHIP1; pTKGhSHIP1m; pTKGhCASP7; pTKGhCASP7m1; pTKGhCASP7m2; pTKGhCASP7m1+2; pTKGhNKX3; pTKGhNKX3m. Both strands of PCR products were sequenced to test for accuracy of mutation.

### Stem-Loop 3’ UTR RACE PCR (SLURP)

After DNase digestion (as mentioned above) SLURP reverse transcription of mRNA was performed using 2.5 µM 'SLURP rt' primer following the protocol described above. The reaction was started at 42 °C for 1 h followed by an inactivation step at 70 °C for 10 min. PCR amplification was carried out using 2.5 U Immolase DNA Polymerase with additional 2x PolyMate Additive (Bioline GmbH), 1 µl (500-700 ng) of cDNA pool, 3 mM MgCl_2_, 0.4 mM dNTPs, 0.4 µM of each primer and 10x buffer in 25 µl total volume.

The desired 3’ UTR sequence was obtained by means of a nested PCR protocol using the primer 'fw i' binding at the 5’ end of the cDNA provided by the 'SLURP rt' primer and a first gene specific primer (GSP) at the 3’ end of the 3’ UTR (GSP rev i). A second round of the nested PCR was performed to enhance specificity and product yield using inlying primers 'fw ii' that bind additional sites within the introduced 'SLURP rt' and 'GSP rev ii'. Both reactions of nested PCR started with initial denaturing at 95 °C for 10 min and only employed 'GSP rev i' or 'GSP rev ii', respectively. Linear amplification was conducted by running 10 initial cycles consisting of denaturing for 30 sec at 95 °C, annealing for 30 sec at 58-62 °C, and elongating for 1-2.5 min at 72 °C. Subsequently, either 'fw i' or 'fw ii' were added to the reaction and 30 cycles of denaturing, annealing and elongation were performed according to the first 10 cycles. The second round of the nested PCR was performed using 0.5-1 µl of the first reaction as a template. Both strands of PCR products were sequenced using GSP primers directly or primer walking after cloning.

## Results and Discussion

### Analysis of miRNA-target interaction by Seed Mutagenesis Assembly PCR (SMAP)

DNA manipulation by site directed mutagenesis and assembly via overlapping PCR termini is a well established approach [15] for altering the genetic code or analysing *cis* regulatory elements by fragment substitution or insertion. Although miRNA interaction with respective target mRNAs relies on incomplete complementarity, Watson-Crick pairing of the nucleotides 2-8 (seed) is regarded to be essential for miRNA mediated silencing [16]. Consequently, mutagenesis of the seed sequence within a predicted miRNA target site is a commonly accepted strategy to evaluate bona-fide miRNA-target interaction. Therefore, we have established a protocol for mutagenesis of seed sequences based on site directed mutagenesis and assembly of mutated termini. Since several target sites of a particular or multiple miRNAs may occur within the 3’ UTR the approach is fully applicable to perform sequential mutagenesis of multiple target sites allowing for structural analysis. For evaluation of the presented approach, we first considered the known interaction between SHIP1 and miR-155 [17]. After template generation using specific primers for the SHIP1 3’ UTR (Figure 1 A) the extended seed sequence was mutated by performing two individual PCR reactions enabling site directed mutagenesis. The seed location was chosen to generate overlapping amplicon termini in each reaction (Figure 1 B) by means of oligonucleotides including mutated seed sequences. For this purpose, two complementary oligonucleotides were employed in each PCR possessing about 12 nucleotides at the 3’ as well as 5’ end that flanked the mutated seed region. This design turned out to optimally stabilise the mutating primers in the PCR providing efficient seed mutagenesis and was applied as a general principle. We also developed a PCR protocol promoting specific and efficient mutagenesis (see amplicon I and II in Figure 1 B). The reaction was composed of two successive basic conditions. Initial 10 cycles were run applying decreased annealing temperatures (54-58 °C gradient +dT 0.5 °C per cycle), which allowed for proper binding of the mutating oligonucleotides (Figure 1 B, geneX-SDMr fw or -f rev) and generation of first products harbouring the terminus with altered seed sequence. The annealing temperature was increased for the following 30 cycles (59-63 °C gradient), guaranteeing targeted amplification of the specific product. As shown by the gel images in Figure 1 B, the protocol generated mutated products with nearly complete absence of by-products. The assembly of mutated and overlapping ends was performed by employing a PCR protocol composed of two successive steps. Within the first step (10 cycles), both amplicons were assembled using low concentrations of each product (0.5 pmol). The second step was succeeded by adding terminal primers flanking the full amplicon (Figure 1 C) and providing 5’ overhangs introducing restriction sites for cloning in the luciferase reporter vector pTKGluc. Resulting plasmids harboured either the mutated (pTKGhSHIP1m) or the wild type (pTKGhSHIP1) SHIP1 3’ UTR and were fused with the 3’ end of the *Gaussia* reporter gene as well as co-transfected into HeLa cells with miR-155 mimics or non-targeting controls to evaluate interaction. As shown in Figure 1 C, miR-155 caused approximately 30% down-regulation of luciferase activity when fused to the wild type SHIP1 3’UTR and compared with the nonsense transfected control. Seed mutagenesis resulted in no difference in luciferase activity after miR-155 as well as nonsense control transfection verifying not only the miR-155-SHIP1 interaction but also validating the proposed protocol.

### NKX3.1 Is a miR-155 Target

**Figure 2:**
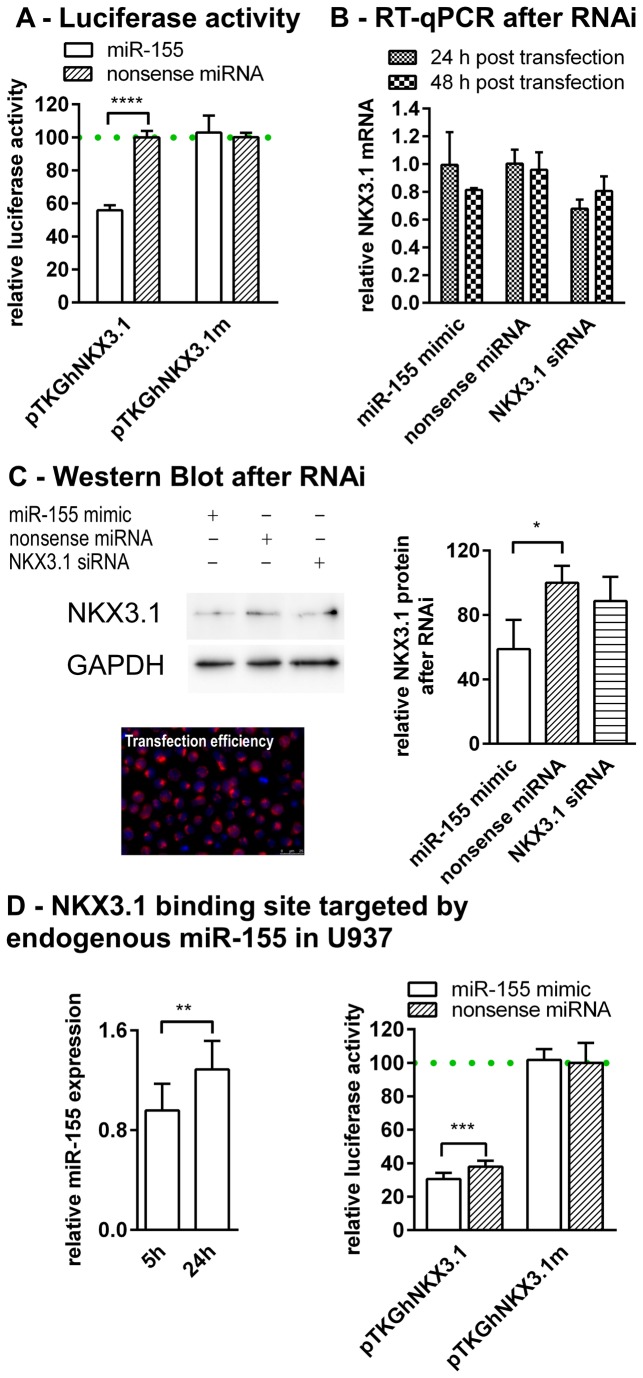
SMAP reveals the transcription factor NKX3.1 as a miR-155 target. Section A: Luciferase reporter assays after mutagenesis proved NKX3.1 as a miR-155 target. Relative luciferase activity (Luc _Gaussia_ : Luc _Cypridina_) was determined using a miR-155 mimic compared with a nonsense miRNA control together with the mutated seed (pTKGhNKX3.1m) as well as wild type control (pTKGhNKX3.1) . The columns show means of normalised luciferase activity of three biological replicates each measured in triplicates while error bars show the standard deviation. Asterisks indicate statistical significance between samples (****: P < 0.001, unpaired t test). Section B: mRNA degradation assays by means of RT-qPCR experiments are shown detecting relative NKX3.1 levels (reference gene: GAPDH). U937 were transfected with miR-155, nonsense miRNA and NKX3.1 siRNA. RNA was isolated at 24 and 48 h post transfection. Columns show mean relative NKX3.1 transcript levels (± SD) of three biological replicates each measured in triplicates compared with nonsense transfected controls. Section C: Western Blots detecting NKX3.1 and GAPDH (reference protein) are shown using the monocytic U937 cells transfected with miR-155, nonsense miRNA and NKX3.1 siRNA. Intrinsic NKX3.1 levels are decreased after miR-155 transfection compared with nonsense controls and siRNA (48 h post transfection). The bar graph shows the luminescence-based relative quantification of protein (NKX3.1:GAPDH) of three individual biological replicates while error bars show the standard deviation. Asterisks indicate statistical significance between samples (*: P < 0.05, unpaired t test). Transfection efficiency was evaluated by transfecting Cy3 labelled nonsense siRNA and fluorescence microscopy. While NKX3.1 translation is markedly inhibited by miR-155, cellular NKX3.1 mRNA levels remained stable after miR-155 at 24 h and decreased after 48 h suggesting slow cellular turnover rates of miR-155 directed NKX3.1 inhibition. Section D: Endogenous miR-155 expression leads to decreased luciferase activity of transfected reporters. The bar graph on the left hand side shows relative miR-155 expression in U937 cells at 5 and 24 h after LPS stimulation. The other bar graph shows that endogenous miR-155 expression results in clear repression of luciferase activity (nonsense transfected) while ectopically introduced miR-155 results in elevated repression and mutated controls remain unaffected. The columns show means of three biological replicates each measured in triplicates while error bars show the standard deviation. Asterisks indicate statistical significance between samples (**: P < 0.01,***: P < 0.001, unpaired t test).

In vivo and in vitro overexpression of miR-155 represses SHIP1 in hematopoietic cells, resulting in increased activation of AKT/PKB after LPS stimulation [17]. SHIP1 is a repressor of PI3K mediated AKT/PKB activation via hydrolysis of the second messenger phosphatidylinositol P3 (PIP3). It inactivates PI(3–5)P3 by hydrolysis to PI(4,5)P2 [18]. Another PI3K antagonist is PTEN, which has a similar effect on AKT/PKB inhibition via its 5’ phosphatase activity [19]. Loss of PTEN in a prostate cancer model was shown to be based on loss of the homeobox-containing transcription factor NKX3.1, resulting in AKT/PKB activation [20]. Since NKX3.1 is also expressed in monocytic cells (Figure 2 B and C) we speculated that apart from SHIP1 it may play a PTEN dependent role on AKT/PKB signalling. Interestingly, in search of further potential miR-155 targets that may regulate AKT/PKB signalling, our in silico analyses predicted NKX3.1 to be a potential target of miR-155. We identified one miR-155 target site within its 3’ UTR possessing an extended seed sequence with an adenosine across position 1 and a uridine across position 9 (Figure 1 A), which according to the studies of Fabian et al. indicates enhanced interaction and activity [4]. NKX3.1 is expressed during embryonic development but also in adult prostate tissue; its loss is related to prostate cancer [21,22]. More recently, it was shown that NKX3.1 mediates proliferation of TAL1 expressing human T cells in acute lymphoblastic leukemia [23]. 

Following the SMAP protocol, we mutated the seed sequence of the identified NKX3.1 target site and fused it to the *Gaussia* luciferase, resulting in the generation of the plasmid pTKGhNKX3.1m and the wild type control (pTKGhNKX3.1). Co-transfection of miR-155 with the wild type 3’ UTR (pTKGhNKX3.1) resulted in 45% mean down-regulation of luciferase activity in HeLa cells compared with the nonsense transfected controls. However, extended seed mutagenesis (pTKGhNKX3.1m) resulted in no difference in luciferase activity after miR-155 as well as nonsense control transfection (Figure 2 A). The potent down-regulation of luciferase activity pointed out NKX3.1 to be a mir-155 target. For validation of luciferase reporter assay data, we employed the human moncytic cell line U937 to examine miR-155 interaction with intrinsic NKX3.1 performing RNAi experiments. For this purpose, U937 were transfected with miR-155 mimics while NKX3.1 siRNA as well as nonsense miRNAs were considered as controls. miRNA-target interaction results in silencing based on translational inhibition or mRNA degradation after deadenylation [24]. We first performed mRNA degradation assays after RNAi (by means of RT-qPCR) to find out if cellular NKX3.1 mRNA levels are affected by miR-155 transfection. As shown in Figure 2 B, miR-155 transfection resulted in stable NKX3.1 transcript levels at 24 h post transfection compared with nonsense controls, while mRNA levels decreased to 67% after NKX3.1 specific siRNA transfection compared with nonsense transfected control. After 48 h the mRNA levels were decreased to 81% after miR-155 or 80% after siRNA transfection, respectively (Figure 2 B). To assess the effect of miR-155 on NKX3.1 translational repression, 48 h post transfection cells were lysed accordingly and subjected to Western Blots. As shown in Figure 2 C, miR-155 mimic caused clear reduction of cellular NKX3.1 protein levels compared with the nonsense control while limited knock-down was achieved applying the NKX3.1 specific siRNA. The application of a miR-155 antagonist tended to raise NKX3.1 levels but not significantly (data not shown). Relative quantification of three independent Western Blot experiments resulted in averaged 40% decreased NKX3.1 protein levels compared with nonsense transfected controls (Figure 2 C). Since the SMAP protocol is proposed to evaluate physiological targets of miRNAs we wanted to exclude that the observed down-regulation of luciferase activity resulted from elevated concentrations of miRNA due to transfection. Therefore, endogenous miR-155 levels were considered for down-regulation of luciferase activity. Since it is known that LPS induces miR-155 expression in hematopoietic cells [25], U937 were stimulated with LPS and transfected with respective reporter plasmids (pTKGhNKX3.1 and pTKGhNKX3.1m) and nonsense miRNA as well as miR-155. This experiment clearly showed that in nonsense transfected controls endogenous miR-155 expression supressed the luciferase activity markedly; while miR-155 transfection had synergistic effects compared with SMAP based mutated controls (Figure 2 D). This approach is in accordance with the studies of Wang et al. [26] who suggested that induction of endogenous miRNAs has possible advantage of more accurately representing genuine miRNA-directed repression. Consequently, our experiments turned out NKX3.1 to be a genuine miR-155 target because reporter assays showed decreased luciferase activity after miR-155-NKX3.1 interaction as well as intrinsic NKX3.1 protein levels were decreased after miR-155 transfection. As a result, we conclude that miR-155 mediated silencing of NKX3.1 in U937 cells seems to predominantly rely on translational inhibition closely linked to a delayed mRNA degradation. This suggests that miR-155 causes moderate cellular mRNA turnover rates of NKX3.1 in monocytic cells when compared with the specific siRNA, which showed a clear degradation right after 24 h. As mentioned above, miR-155 functions as an AKT/PKB activator in hematopoietic cells by down-regulation of the phosphatase SHIP1. Moreover, the loss of another negative regulator of AKT/PKB signalling, the phosphatase PTEN, is based on lacking NKX3.1. Silencing of SHIP1, on one hand, as well as NKX3.1 downregulation leading to PTEN inhibition, on the other hand, suggest synergistic effects resulting in potent activation of AKT/PKB signalling by miR-155 in monocytic cells. Our on-going studies will decipher the role of NKX3.1 in mentioned signalling pathways.

### Sequential seed mutagenesis by SMAP revealed one of two predicted target sites to confer predominant interaction of miR-29a and its target CASP7 

For determination of new miRNA-mRNA interactions we apply a strategy based on employing a series of target prediction and pathway analysis tools for computational identification of most probable targets. This protocol relies on initial target prediction of selected miRNAs of interest using the tool Target Scan [9]. The generated list of hypothetical targets is used as a gene list to enter a bioinformatics database such as DAVID [27] and trace potentially affected pathways. DAVID has the benefit of combining several pathway databases such as KEGG or Biocarta. Dependent on the chosen pathway database, this step allows for selection of pathway accumulated targets of interest (e.g. after treatment, disease etc.) because targeting of multiple components of a signalling pathway by a given miRNA would indicate robustness of predication. The miRNA-mRNA interaction between identified targets is evaluated using the tool RNAhybrid, which tests for hybridisation characteristics between a given miRNA and its target mRNA [11]. The presented workflow allows data filtering and narrowing down of the number of predicted targets concentrating on most probable interactions.

**Figure 3:**
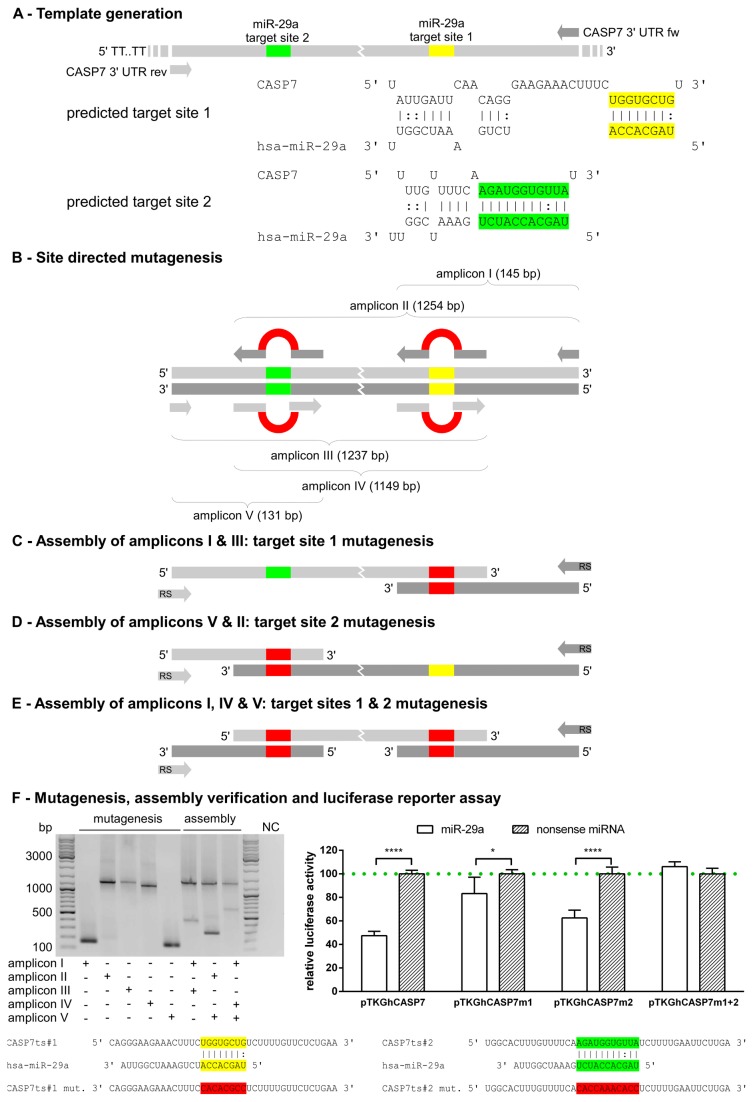
Sequential mutagenesis of multiple miR-29a target sites within the 3’ UTR of CASP7 by means of SMAP. Section A: Full length 3’ UTR of CASP7 was generated harbouring both identified target sites of miR-29a. Section B: For individual but also combined target site mutagenesis, the 3’ UTR was theoretically divided in three segments defined by locations of target sites. Sections C-E: Modular assembly of amplicons resulted in sequential mutagenesis of target sites. Section F: Gel image shows five amplicons after mutagenesis as well as assembled products for reporter gene fusion. NC represents the no template control of the assembly PCR. Relative luciferase activity (Luc _Gaussia_ : Luc _Cypridina_) was determined using a miR-29a mimic compared with a nonsense miRNA control together with the mutated target site 1 (pTKGhCASP7m1), mutated target site 2 (pTKGhCASP7m2), mutated target sites 1 and 2 (pTKGhCASP7m1+2) as well as the wild type control (pTKGhCASP7) . The columns show means of normalised luciferase activity of three biological replicates each measured in triplicates while error bars show the standard deviation. Asterisks indicate statistical significance between samples (*: P < 0.05; ***: P < 0.001, paired t test).

Based on integrative analysis of miRNA and mRNA transcriptomes after mycobacterial infections and following the workflow described above, we were able to define CASP7 as a miR-29a target [14]. In this previous study, we predicted two potential target sites within the CASP7 3’ UTR (Figure 3), however no further structural analysis of target site impact on miR-29a mediated CASP7 regulation has been performed. Based on the established SMAP protocol, we sequentially mutated each target site in the present study to evaluate target site impact on miR-29a mediated CASP7 regulation. For individual but also combined target site mutagenesis, the CASP7 3’ UTR was theoretically divided in three segments defined by location of predicted target sites as well as 5’ and 3’ termini of the 3’ UTR. The locations of each seed were taken as binding sites for mutagenesis primers providing overlapping termini as described above (Figure 3 A). As shown in Figure 3 B, this design allowed the generation of 5 amplicons either mutating target site #1 (in amplicons I and III) or target site #2 (in amplicons II and V) or both target sites (in amplicon IV). Full length 3’ UTRs with individually mutated seed sequences were obtained after assembly of amplicons I and III (Figure 3 C) or II and V (Figure 3 D), respectively. On the other hand, the assembly of amplicons I, IV and V in one reaction resulted in a full length 3’ UTR containing both mutated seeds (Figure 3 E). As shown in Figure 3 F, the developed PCR regime resulted again in amplification of specific bands both for mutagenesis and assembly. Each of the three assembled amplicons as well as the wild type 3’ UTR were fused to the *Gaussia* luciferase as described above providing the following reporter plasmids for structural analysis of target sites: pTKGhCASP7 (control), pTKGhCASP7m1 (mutated seed 1), pTKGhCASP7m2 (mutated seed 2) and pTKGhCASP7m1+2 (mutated seeds 1 and 2).

Successive luciferase reporter assays using the wild type 3’ UTR (pTKGhCASP7) showed more than 50% down-regulation of luciferase activity when co-transfected with miR-29a compared with nonsense controls (Figure 3 F). Interestingly, this modular approach showed that mutagenesis of target site #1 resulted in less than 20% mean down-regulation of luciferase activity. On the other hand, alteration of the seed sequence within target site #2 produced an approximate 40% decrease of luciferase activity while there was no difference between miR-29a and nonsense transfected controls when target sites were mutually mutated (Figure 3 F). This experiment revealed target site #1 to be the main *cis* regulatory element for miR-29a mediated CASP7 regulation. This is supported by the fact that Target Scan analysis of miR-29a-CASP7 interaction showed only conservation of target site #1 among mammals but not target site #2. On one hand, target site #1 mutagenesis did not restore the nonsense control condition and alteration of the seed sequence within target site #2 did not result in full magnitude of down-regulation. On the other hand, mutual mutagenesis resulted in no effect between miR-29a and nonsense transfection. As a result, we conclude that synergistic effects of both sites exist. This is interesting, since target site #2 is a non-canonical miRNA target site with one G:U wobble within the seed sequence and without central stretches of non-complementary regions possessing 90% complementarity to miR-29a. Because it features only a single mismatch across the position 12 (not possessing a central bulge) of miR-29a (Figure 3 A) we mutated the extended seed sequence between nucleotide 1 and 11. Interestingly, studies e.g. of *Caenorhabditis elegans* have shown that perfect Watson-Crick pairing along the seed is not a fundamental condition for miRNA mediated silencing [7]. Also more recently it was shown that G-bulge sites at positions 5 or 6 of seeds are bound by miR-124 in the mouse brain and confer efficient silencing [6]. The identified target site #2 within the CASP7 3’ UTR is conserved between human and Chimpanzee but not Rhesus, which suggests novel evolutionary acquisition in higher primates that potentially confers effective CASP7 repression through synergistic effects in *Hominidae*. This hypothesis is based on the knowledge that multiple sites within the same 3’ UTR for the same or different miRNAs are needed for enhanced inhibitory activity [5]. Apart from these findings, our study on the CASP7-miR-29a interaction convincingly showed that observed effects on luciferase activity result from number and nature of sites rather than elevated concentrations of miRNA and putative target sequences due to transfection. Taken together, it points out the SMAP protocol as a tool for identification and validation of physiological targets of miRNAs.

### Rapid identification of 3’ UTR sequences by means of Stem-Loop 3’ UTR RACE PCR (SLURP)

**Figure 4:**
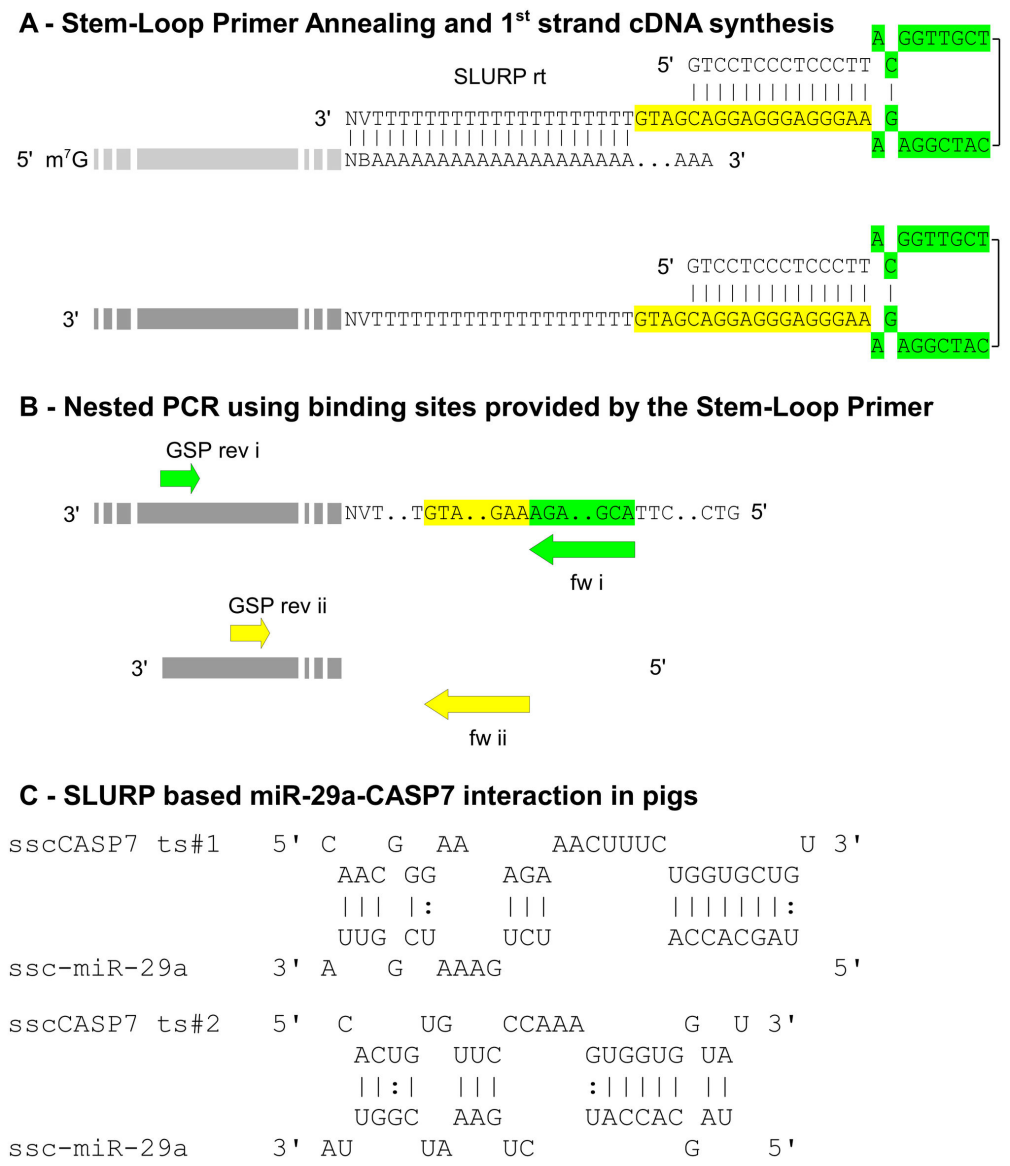
Rapid identification of 3’ UTR sequences by means of Stem-Loop 3’ UTR RACE PCR (SLURP). Section A: Figure shows stem-loop primer (SLURP rt) binding and priming of the reverse transcription reaction. As highlighted in green and yellow, the 'SLURP rt' provides two primer binding sites for a nested PCR that are hidden by the secondary structure. Section B: The use of two batched gene specific primers (GSP rev i and GSP rev ii) together with 'fw i and fw ii' binding to the sites provided by the 'SLURP rt' allowed for a nested PCR. Section C: Identified miR-29a target sites within the 3’ UTR of porcine CASP7. SLURP based sequence revealed that target site 1 was highly conserved between humans and pigs. The predicted target site 2 suggested non-canonical binding of miR-29a as also shown in humans.

In recent years we have focused our studies on miRNA regulation of post natal intestinal development and disease using piglets as a model [3,28]. In contrast to humans or mice, in non-model organisms such as pigs sequence data on untranslated mRNA regions are either entirely or partly lacking. Since these sequences are needed for prediction of miRNA-mRNA interactions, we were confronted with the problem of generating de novo 3’ UTR sequences by means of RACE experiments. For straightforward and eased investigation of 3’ UTR sequences, we developed a new RACE protocol called SLURP that is based on employing a stem-loop oligo (dT) primer for cDNA generation. This anchored oligonucleotide consists of a 3’ oligo (dT)_20_ fragment hybridising with the first adenosines of a polyadenylated RNA molecule followed by a 5’ part, which provides two primer binding sites for a nested PCR hidden in a stem-loop secondary structure. The secondary structure is stabilised by a stem region composed of 14 complementary nucleotides having a calculated T_M_=47 °C and a dG=-22.6 kc/m. This avoids double-strand dissociation during cDNA synthesis. The use of an anchored stem-loop oligonucleotide harbouring primer binding sites for subsequent nested PCR avoids mispriming since binding sites are blocked during first strand synthesis by means of stem-loop formation (Figure 4 A). Increased temperature during PCR unfolds the secondary structure making the binding sites accessible. The use of two batched gene specific reverse primers (GSP rev i and ii, Figure 4 B) together with two forward primers binding to the region provided by the stem-loop primer, allowed a nested PCR approach for enhanced specificity and product yield (Figure 4 B). A PCR protocol based on linear amplification over the initial 10 cycles followed by 30 cycles of exponential amplification turned out to result in enhanced specificity of the approach. For this purpose, the first 10 cycles were performed by only adding the GSP rev i (or for the 2^nd^ round of the nested PCR GSP rev ii) followed by addition of the fw i primer (or for the fw ii for the 2^nd^ round of the nested PCR) for the remaining 30 cycles. In this study, SLURP was used to identify the sequences of four porcine 3’ UTRs that were chosen according to our research foci. Based on partially known mRNA sequences, we determined full length 3’ UTR of DHH (445 bp, Accession No. HE651025), SELPLG (768 bp, Accession No. HE651024), TGFBR3 (1378 bp, Accession No. HF566399) and CASP7 (570 bp, Accession No. HF566398). 

As mentioned above, we have already shown that CASP7 is regulated by miR-29a in primary human macrophages [14]. In another study using pigs as a model, we determined that miR-29a mediated CAV2 regulation leads to increased cellular levels of active CDC42 [28]. Interestingly, it is known that CDC42 is cleaved by CASP7 and CASP3, but not by other caspases such as 6 and 8 [29]. As a result, we became interested in examining similarities between human and porcine binding sites within the CASP7 3’ UTR, although the analysis of CASP7 binding site #2 for miR-29a revealed no conservation among primates. Since relating porcine sequence data relied either on computational predictions (XM_001928978) or partial mRNA data (AK233348) we decided to re-sequence the porcine CASP7 3’ UTR employing the SLURP protocol. Porcine target site #1 was highly conserved compared with the respective human binding site possessing 89% sequence identity (Figure 4 C) and complete seed complementarity. However, porcine target site #2 (Figure 4 C) showed only 76% identity between pigs and humans and exhibited one mismatch across the 3^rd^ nucleotide of miR-29a. Although CASP7 target site #2 is a non-canonical binding site characterised by the mismatched nucleotide in the seed region, it is known that such sites can still confer inhibition and that potent silencing of target mRNAs by miRNAs relies not only on target site nature, but also on the number and accessibility of binding sites [5]. Consequently, 3’ UTR secondary structures affect inhibition properties by determining the accessibility of target sites [30]. Our studies suggest that sole fusion of the target site (for example realised by hybridised oligonucleotides) to a reporter gene only partially resembles the inhibition mechanisms since secondary structure formation of 3’ UTRs are not considered. Therefore, we suggest employing long 3’ UTR fragments for reporter gene fusions including mutated controls (generated by SMAP) to mimic molecular interactions as it considers *cis* regulatory elements as well as site accessibility. The structural analysis of miRNA-mRNA interplay in hitherto unknown mRNA sequences as presented here allows for examining the interaction capability of a given site by means of seed mutagenesis. In addition, sequential seed mutagenesis enables distinction between the activities of multiple binding sites within the same 3’ UTR. At the same time, binding site accessibility is resembled based on secondary structure formation of 3’ UTR since long fragments including mutated seeds are compared with wild type 3’ UTRs.

## Conclusions

Interaction between microRNAs and mRNAs has shed light on the regulation of many cellular pathways in development and disease. Functional analysis of predicted interactions employing straightforward protocols as presented here is a fundamental step to decipher mechanisms. The developed approach for microRNA target site mutagenesis based on nucleic acids synthesis strategies, allows for structural analysis of multiple microRNA target sites of a given mRNA. This innovation is of great interest, since effective regulation of a particular mRNA requires multiple target sites of the same or different microRNAs. The integrative strength of presented technologies relies on combination of 3’ UTR sequence identification and functional target analysis in hitherto unknown data, facilitating discovery of regulative pathways also in non-model organisms.
